# Multi-Responsive Nanocarriers Based on β-CD-PNIPAM Star Polymer Coated MSN-SS-Fc Composite Particles

**DOI:** 10.3390/polym11101716

**Published:** 2019-10-19

**Authors:** Feng Guo, Guiying Li, Songmei Ma, Hengquan Zhou, Xinyi Chen

**Affiliations:** College of Chemistry and Materials Science, Ludong University, Yantai 264025, China; guofengpolymer@126.com (F.G.); 13256627828@126.com (S.M.); 15553595198@163.com (H.Z.); ligyingying@163.com (X.C.)

**Keywords:** disulfide bond, ferrocene, multi-responsive, nanocarrier, MSNs

## Abstract

A temperature, glutathione (GSH), and H_2_O_2_ multi-responsive composite nanocarrier (MSN-SS-Fc@β-CD-PNIPAM) based on β-cyclodextrin-poly(*N*-isopropylacrylamide) (β-CD-PNIPAM) star polymer capped ferrocene modified mesoporous silica nanoparticles (MSN-SS-Fc) was successfully prepared. The surface of the mesoporous silica was first modified by ferrocene (Fc) via a disulfide bond (–SS–) to form an oxidizing and reducing site and then complexed with a β-CD-PNIPAM star shaped polymer through host–guest interactions as a nano-valve to provide temperature responsive characteristics. The structure and properties of the complex nanoparticles were studied by FTIR, TGA, EDS, Zeta potential, and elemental analysis. Doxorubicin (DOX) and Naproxen (NAP), as model drugs, were loaded into nanocarriers to assess drug loading and release behaviour. The release of drugs from nanocarriers was enhanced with an increase of the GSH, H_2_O_2_ concentration, or temperatures of the solution. The kinetics of the release process were studied using different models. This nanocarrier presents successful multi-stimuli responsive drug delivery in optimal stimuli and provides potential applications for clinical treatment.

## 1. Introduction

Drug delivery vehicles with stable structures and high biocompatibility must be developed to meet the needs of clinical treatments for pathological environments. A variety of nanocarriers, including cationic materials, recombinant protein materials, liposomes, and vesicles, have been applied to enhance the stability of guest molecules [1,2,3]. Moreover, mesoporous silica nanoparticles (MSNs) with controllable morphology and huge specific surface areas have been widely used as drug carriers in the last few decades [4,5,6]. These nanoparticles exhibit three-stage degradation behavior in the body and can be excreted by feces or urine without changing the microstructures of the kidneys. There are abundant silicon hydroxyl groups (Si–OH) on the surface and inside the channels of MSNs, which provide the possibility for further modification of the mesoporous silica. Inorganic mesoporous silica and the functional organic material can be bridged by a silane coupling agent to encapsulate guests [7,8,9,10,11,12]. For example, Chen et al. designed an intelligent pH-responsive drug release system that uses hyaluronic acid as a blocking agent to encapsulate drugs in MSNs via hydrazine bonds [13]. Hyaluronic acid on the surface of MSNs could also be used as a targeting agent to recognize CD44 overexpressing cells, and hydrazine bonds could be hydrolyzed to release drugs under weakly acidic conditions.

The principle of radiotherapy and chemotherapy is to induce a large amount of oxygen free radicals inside cancer to kill tumor cells, while a small amount of oxygen free radicals promote cells proliferation and growth [14]. Cancer cells often produce multi-drug resistance due to the abnormal increase of glutathione (GSH) content in the environment when the intermittent stimulation of drugs is gradually increased along with the treatment [15,16,17]. Oxidative stress promotes the proliferation and high expression of GSH levels, which confer an antioxidant capacity to tumor cells and make the chemotherapy effect function below expectations due to this delicate balance. Compared with normal tissues, the tumor intracellular environment shows an acidic pH in the endosome and lysosome (5.0–5.5), a high GSH concentration, and an oxidative microenvironment in the mitochondria due to a high concentration of hydrogen peroxide (H_2_O_2_). Many stimuli-sensitive drug delivery carriers have been constructed from MSNs, which can be stimulated by GSH stimulating factors from tumor tissues. For instance, mesoporous silica nanoparticles were degraded by GSH because they contain disulfide linkages (S–S) with polyethylene glycol (PEG) on the outer shell or because transferrin was grafted on the surface of the MSN via S–S simultaneously serving as a capping agent and targeting ligand to transport drugs [18,19]. However, the trigger condition of those vehicles was single and cannot satisfy the complex microenvironment in tumor cells. Multi-sensitive nanocarriers triggered by reducing GSH and oxidant H_2_O_2_ have been seldom reported.

**Scheme 1:**
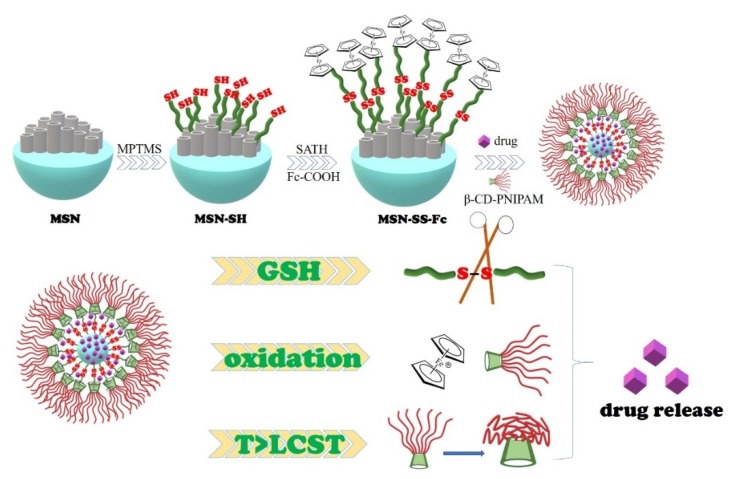
Formation of MSN-SS-Fc@β-CD-PNIPAM nanocarriers and a schematic illustration of drug release.

In this paper, a multi-stimuli sensitive drug delivery system was constructed from mesoporous silica and temperature-sensitive polymers, which can be stimulated by GSH, H_2_O_2_, and temperatures from tumor tissues. These nanocarriers were designed based on the host–guest inclusion interactions between ferrocene modified mesoporous silica through disulfide bonds and the multi-arm star polymer β-CD-PNIPAM, as shown in Scheme 1. Overexpressed GSH and H_2_O_2_ in tumor cells can reduce disulfide bonds (–SS–) to sulfhydryl groups (–SH) and oxidize ferrocene (Fc) to ferrocene ions (Fc^+^), respectively, both of which can destroy the structure of nanocarriers [20,21,22]. The temperature-sensitive PNIPAM modified cyclodextrin star polymer was introduced into the surface of the nanocarriers as a nano-valve and responded to the high temperature of the tumor microenvironment. 

## 2. Experimental Section

### 2.1. Materials

The (3-Mercaptopropyl)trimethoxysilane (MPTMS) and L-Glutathione reduced (GSH) were provided from Macklin Biochemical Co. Ltd. (Shanghai, China). The tetraethyl orthosilicate (TEOS) (Damao Chemical Reagent Co. Ltd, Tianjin, China), cetyltrimethylammonium bromide (CTAB) (Damao Chemical Reagent Co. Ltd, Tianjin, China) and β-cyclodextrin (β-CD) (Shanghai Chemical Reagent Company, Shanghai, China) were analytical grade. Ferrocenedicarboxylic acid (Fc-COOH), doxorubicin (DOX), D,L-Naproxen (NAP), 1-ethyl-3-(3-dimethylaminopropyl)carbodiimide hydrochloride (EDC), and *N*-hydroxysuccinimide (NHS) were provided from Civi Chemical Technology Co. Ltd. (Shanghai, China). *N*-isopropylacrylamide (NIPAM) was purchased from J&K Chemical Ltd. (Beijing, China); 2,2-Dithiodipyridine and cysteamine hydrochloride were purchased from Aladdin Industrial Co. Ltd. (Shanghai, China). All reagents were used without further purification.

S-(2-Aminoethylthio)-2-thiopyridine hydrochloride (SATH) was synthesized according to a previous report [23]. H NMR (CD_3_OD as an internal standard) δ 3.02 (2H, t), 3.27 (2H, t), 7.25 (1H, t), 7.65 (1H, d), 7.75 (1H, t), 8.37 (1H, d).

The star polymer β-cyclodextrin-poly(*N*-isopropylacrylamide) (β-CD-PNIPAM) was prepared by atom transfer radical polymerization (ATRP) with a yield of 65.27% according to a previous report [7]. The arm number of β-CD-PNIPAM was calculated to be 7, and the repeating units of PNIPAM per arm were about 21 based on their ^1^H NMR spectrum. The average molecular weight of the β-CD-PNIPAM star polymer was calculated to be 19,950 g/mol.

### 2.2. Measurements

Fourier transform infrared spectrum (FTIR) analysis was performed on a Nicolet Magna-IR 550 FTIR spectrometer (Nicolet, Madison, WI, USA) in the range of 4000–400 cm^−1^. Transmission electron microscopy (TEM) and scanning electron microscopy (SEM) were recorded with a JEOL JEM-1230 transmission electron microscope (JEOL, Tokyo, Japan) and Hitachi SU8010 scanning electron microscope (Hitachi, Tokyo, Japan), respectively. Zeta potential was determined by a laser particle analyzer using a Malvern Nano ZS90 laser particle analyzer (Malvern, Worcestershire, UK). Thermogravimetric analysis (TGA) was obtained by a Netzsch STA409PC (Netzsch, Wunsiedel, Germany) from 25 to 800 °C with a heating rate of 10 °C/min under N_2_. The UV–Vis absorbance spectrum was carried out by a Shimadzu UV-2550 spectrophotometer (Shimadzu, Kyoto, Japan). The chemical composition of the samples was obtained by an Elementar vario EL cube elemental analyzer (Elementar, Frankfurt, Germany). Nuclear magnetic resonance (NMR) spectrum analysis was performed on a Bruker AVANCE NEO 500-MHz nuclear magnetic resonance instrument (Bruker, karlsruhe, Germany). 

### 2.3. Preparation of Ferrocene Modified Mesoporous Silica Nanoparticles

#### 2.3.1. Preparation of MSNs

A mixture of CTAB (1.00 g) and NaOH (3.50 mL, 2.0 M) was added to 480 mL deionized water with mechanical stirring at 80 °C for 1 h. Then, TEOS (5.00 mL) was slowly added into the mixture at 80 °C with vigorous mechanical stirring for another 2 h. The solid product was centrifuged and washed with deionized water and methanol. To remove the CTAB template, crude products (MSNs) was calcined at 550 °C for 6 h.

#### 2.3.2. Synthesis of MSN-SH

MSNs (0.27 mg) were activated under N_2_ at 170 °C for 10 h and then suspended in MeOH (60 mL). MPTMS (5.5 mL) was added in the above mixture at reflux for 24 h at 55 °C under N_2_. The resulting product (MSN-SH) was obtained by centrifugation (9.5 krpm, 20 min), washed five times with methanol, and then dried in vacuo at 60 °C overnight.

#### 2.3.3. Synthesis of MSN-SS-NH_2_

Synthesis of the disulfide bonds on the surface of the nanoparticles was achieved by suspending the MSN-SH (200 mg) in MeOH (30 mL) and adding SATH (200 mg). The mixture was stirred at room temperature for 24 h under N_2_. The solid was collected by centrifugation (9.5 krpm, 20 min) and thoroughly washed with MeOH and H_2_O. Then MSN-SS-NH_2_ was dried under a vacuum at 80 °C for 24 h.

#### 2.3.4. Synthesis of MSN-SS-Fc

The dried MSN-SS-NH_2_ (0.10 g) was added into the DMF (10 mL) solution containing Fc-COOH (0.03 g), NHS (0.045 g), and EDC (0.09 g). The mixture was stirred at room temperature for 48 h under an N_2_ atmosphere. The resulting product (MSN-SS-Fc) was centrifuged (9.5 krpm, 20 min) and washed several times with methanol. The product was then dried at 50 °C in a vacuum for 12 h. The yield of the MSN-SS-Fc was 81.6%

### 2.4. Preparation of MSN-SS-Fc@β-CD-PNIPAM Nanoparticles and Drug Loading

MSN-SS-Fc@β-CD-PNIPAM composite nanoparticles were obtained by adding the same concentration of β-CD-PNIPAM dropwise into the MSN-SS-Fc solution with stirring for 24 h. Drug-loaded nanoparticles were prepared by using NAP and DOX as model drugs. Specifically, a certain amount of model drug was dissolved in the MSN-SS-Fc/DMF solution. Then a β-CD-PNIPAM solution (0.4 mg/mL) was added dropwise into the above solution with stirring for 24 h. The dialysis pockets were placed into 150 mL fresh ultrapure water with stirring at room temperature. Fresh ultrapure water was replaced several times during the dialysis process until the drugs could not be detected in the last dialysis. The drug loading content (DLC%) and entrapment efficiency (EE%) of the nanocarriers were calculated according to the following formulas. In order to apply rigorous statistical analyses, three separate experiments were made in each case for averaging:

(1) $$\mathrm{DLC}\%=\frac{m_{0}-cv}{m+m_{0}-cv}\times 100\%$$

(2) $$\mathrm{EE}\%=\frac{m_{0}-cv}{m_{0}}\times 100\%$$

where *m*_0_ is the weight of the drug dosage; *m* is the weight of the nanoparticles; *c* is the concentration of the DOX in the dialysis fluid measured by UV–Vis analysis (mg/L); *v* is the volume of dialysis solutions.

### 2.5. Drug Release from MSN-SS-Fc@β-CD-PNIPAM Nanoparticles

The release behavior was studied by a UV–Vis spectrophotometer. Specifically, drug-loaded nanocarriers (5.0 mL) were transferred into dialysis pockets (MWCO 8-14 kDa, Biosharp) and placed into 50 mL PBS at different temperatures (25, 37, and 42 °C) with different concentration of H_2_O_2_ (0.1%, 0.5%, 1%) or GSH (0, 2, and 10 mM). A 5.0 mL solution containing drugs was taken out for UV–Vis analysis, and an equal volume of fresh PBS was added into the environmental system at given intervals. The cumulative release of the drugs was computed according to the following formula.

(3) $$\mathrm{Cumulative}\;\mathrm{release}=\frac{50C_{n}+5.0\sum C_{n-1}}{m_{0}-cv}$$

where *C_n_* and *C_n_*_−1_ are the concentration of drugs released from the nanocarriers measured via UV–Vis analysis at *n* and *n−*1 times, respectively (mg/L); *n* is the time of removal from the PBS (*n* > 0); other symbols are as the same as those mentioned above.

## 3. Results and Discussion

### 3.1. Synthesis and Characterization of Nanocarriers

**Figure 1:**
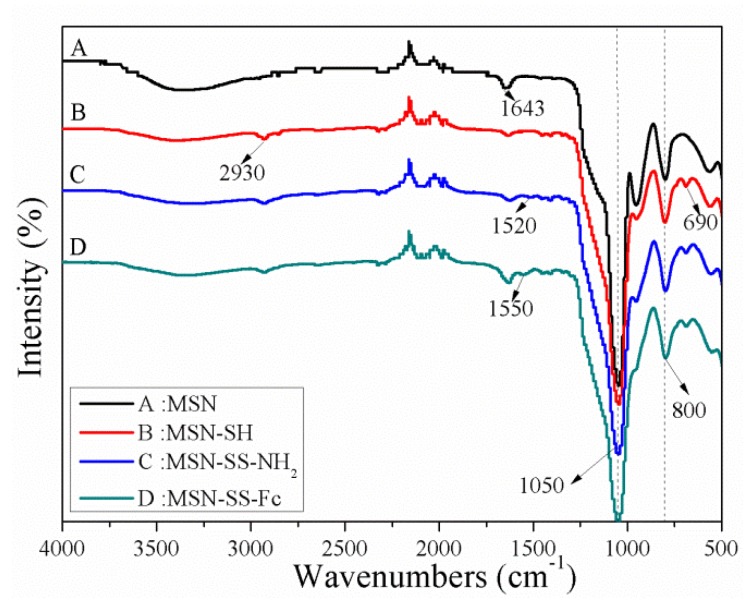
FTIR spectra of MSNs (**A**), MSN-SH (**B**), MSN-SS-NH_2_ (**C**), and MSN-SS-Fc (**D**).

Specifically, the porous MSNs were prepared by using CTAB as hard templates. Inorganic hybrid materials were formed by condensing the TEOS precursor on the micelle′s surface, which finally removed the surfactant by calcining to produce channels. Then MSNs were functionalized with MPTMS, SATH, and Fc-COOH in a sequential process to obtain the final product MSN-SS-Fc. The FTIR spectra of the MSNs and surface modification of MSNs are shown in Figure 1. The two noticeable peaks at 1050 and 800 cm^−^^1^ correspond to the asymmetric and symmetric stretching vibrations of Si–O–Si, respectively. Surface functionalization was confirmed by the weakening of the Si–OH vibration at 950 cm^−1^ and the newly appearing absorption bands at 2930 and 690 cm^−1^, which was associated with the C–H asymmetric stretching vibration and rocking vibration. The reaction of MSN-SH with SATH resulted in the appearance of –NH_2_ bending vibrations at 1520 cm^−1^. The absorption band of the amino group migrated from 1520 to 1550 cm^−1^, which was related to the reaction of Fc-COOH with MSN-SS-NH_2_.

**Figure 2:**
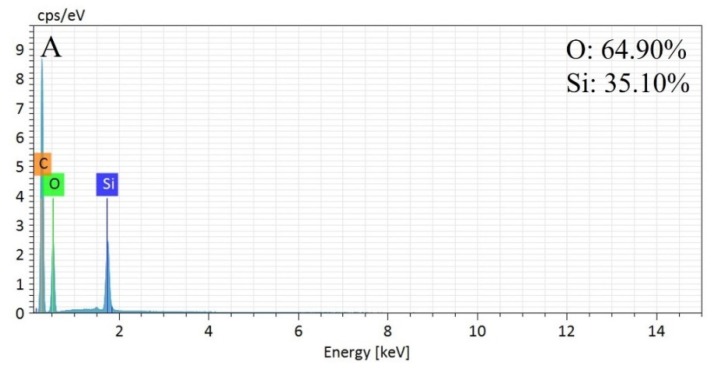

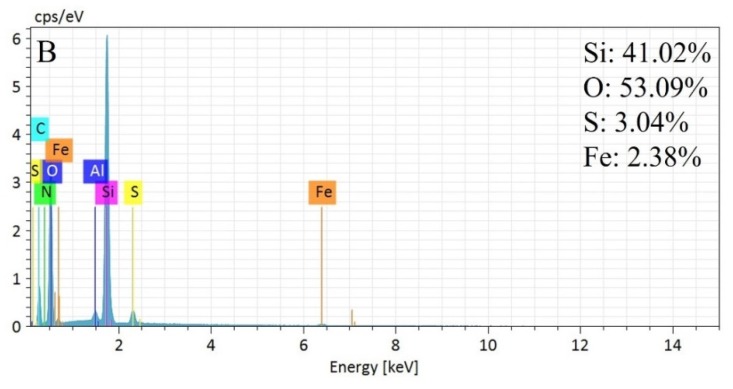
Element distribution spectra for MSNs (**A**) and MSN-SS-Fc (**B**).

Figure 2 shows the element distribution spectra of the MSNs and MSN-SS-Fc from EDS. It can be seen that the Si and O elements were all contained in the MSNs and MSN-SS-Fc. The normalization percentage of the Si and O elements was 35.10% and 64.90%, respectively, in MSNs. For MSN-SS-Fc, the normalization percentage of the elements Si, O, S, and Fe was 41.02%, 53.09%, 3.04%, and 2.38%, respectively. The results indicate the modification of MSNs with ferrocene by disulfide bonds.

The chemical composition of the MSNs, MSN-SH, and MSN-SS-Fc was also studied by elemental analysis, as shown in Table 1. After modification with MPTMS, the C and H contents of MSN-SH increased from 1.69% and 0.71% to 6.23% and 1.40%, respectively. Especially, the content of S (3.58%) indicated that the sulfhydryl group reacted on the surface of the MSNs. Meanwhile, the C and H contents of the ferrocene-functionalized MSN-SS-Fc further increased to 7.77% and 1.86%, respectively.

**Figure 3:**
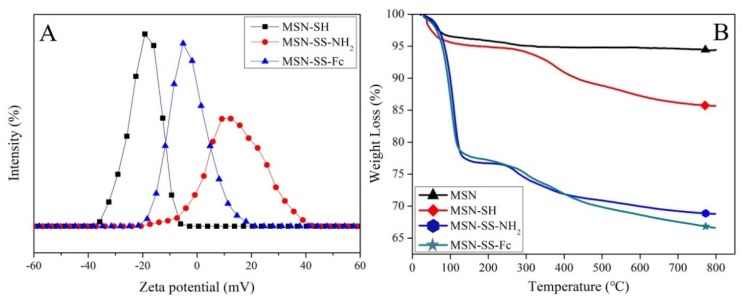
The zeta potentials of MSN-SH, MSN-SS-NH_2_, and MSN-SS-Fc (**A**) and the TGA analysis of MSN, MSN-SH, MSN-SS-NH_2_, and MSN-SS-Fc (**B**).

In addition, modifying the ferrocene on the MSNs via disulfide bonds was successfully confirmed by the variation of the zeta potential value, as shown in Figure 3A. The zeta potential of MSN-SH was −19.6 mV due to the ionizing of sulfhydryl. The zeta potential of MSN-SS-NH_2_ increased from the negative value of −19.6 mV to the positive value of +24.5 mV due to the amine groups connected on the surface of the mesoporous silica. After reacting with Fc-COOH, the zeta potential of MSN-SS-Fc decreased to the electroneutrality value of +0.44 mV.

The TGA curves of MSNs, MSN-SH, MSN-SS-NH2, and MSN-SS-Fc are shown in Figure 3B, clearly indicating the successful modification of the nanoparticles. The initial weight loss of all samples from 50 to 100 °C was due to the physically adsorbed water [24]. Compared to the weight loss of the blank MSNs, the additional weight loss of about 8.72% for MSN-SH was assigned to the removal of the organosilane portion. An increase in weight loss was observed in the TGA curves of MSN-SS-NH_2_ and MSN-SS-Fc. The weight losses percentage of the organic matter in MSN-SS-NH_2_ increased from about 8.72% to 16.89% after SATH grafting on MSNs, and a further 2.18% of organic matter was lost after the modification of ferrocene groups on MSN-SS-NH_2_.

**Figure 4:**
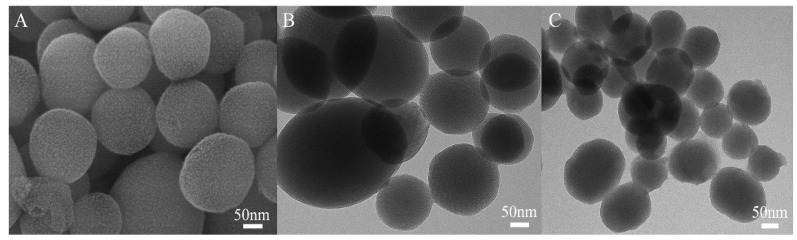
SEM image of MSNs (**A**) and TEM images of MSNs (**B**); MSN-SS-Fc@β-CD-PNIPAM complex nanoparticles (**C**).

The MSN-SS-Fc@β-CD-PNIPAM complex nanoparticles were formed via host–guest interactions between the β-CD-PNIPAM star polymer and MSN-SS-Fc. The microstructures of unmodified MSNs and complex nanoparticles were clearly revealed by SEM and TEM. Figure 4A,B show that the blank MSNs had a well-defined spherical shape with a uniform particle size of 100–150 nm. The morphology of the blank MSNs revealed highly ordered hexagonal array features, as shown in TEM, which indicated their mesoporous structure. Compared to unmodified MSNs, Figure 4C shows the typical TEM image of complex nanoparticles with an unsharp spherical boundary modified by organic materials. Mesoporous silica nanoparticles with this structure can easily cross the blood–brain barrier without side effects [25,26].

**Figure 5:**
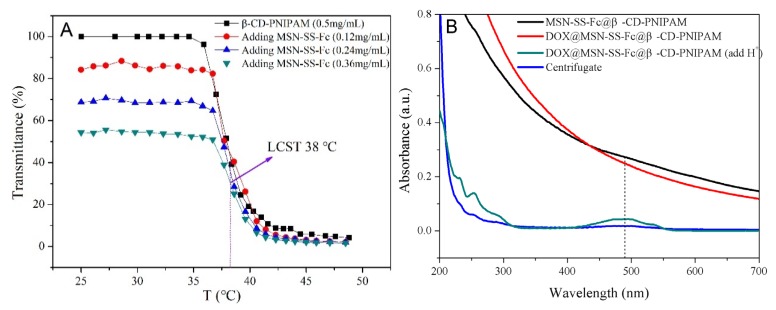
Transmittance of nanoparticles at different temperature (V_β-CD-PNIPAM_: V_MSN-SS-Fc_ = 1:1) (**A**) and the UV–Vis absorption spectra of blank nanoparticles, DOX-loaded nanoparticles; DOX-loaded nanoparticles after adding H^+^ and undergoing centrifugation (9.5 krpm, 20 min) in water (**B**).

The temperature sensitivity of the MSN-SS-Fc@β-CD-PNIPAM composite nanoparticles was proven by the changes in their transmittance at different temperatures from V-1100D spectrophotometer (Shanghai Meipuda instrument Co. Ltd, Shanghai, China). Hydrophilic PNIPAM chains obtain hydrophobicity when their temperature is higher than the lower critical solution temperature (LCST) [27,28]. As shown in Figure 5A, the transmittance of the β-CD-PNIPAM star polymer and complex nanoparticles was all stable at room temperature. With the addition of MSN-SS-Fc in the nanoparticles, the transmittance decreased, suggesting the formation of host–guest interactions between β-CD-PNIPAM and MSN-SS-Fc. As the temperatures increased above 35 °C, the light transmittance of the nanoparticles and β-CD-PNIPAM star polymer showed a distinct decrease due to the transformation of the PNIPAM chains from hydrophilicity to hydrophobicity. It was seen that all curves showed the same LCST at about 38 °C, indicating that the complexation of MSN-SS-Fc does not change the temperature sensitivity of β-CD-PNIPAM.

### 3.2. Encapsulation Drugs in Complex Nanoparticles

To prove the encapsulation capacity of nanocarriers, DOX and NAP as model drugs were loaded into MSN-SS-Fc@β-CD-PNIPAM complex nanocarriers. The DLC% and EE% are shown in Table 2. Especially, the DLC% of nanoparticles for DOX and NAP reached 41.81% and 34.64% with an increase of drug dosage to 100%. The EE% reached the maximum value of 77.85% and 71.23% for DOX and NAP, respectively. The high DLC% of nanoparticles might be attributed to the fact that the drugs can be loaded not only in the channels of mesoporous silica, but also in the cavities of cyclodextrin or attached to nanoparticles through hydrogen bonds or electrostatic interactions [29]. The star polymer β-CD-PNIPAM capped onto the surface of MSNs prevented the premature leakage of guest molecules from nanocarriers. The UV–Vis spectra in Figure 5B were developed to evaluate the successful loading of drugs into the nanoparticles. The solution of blank nanoparticles and DOX loaded nanoparticles after centrifugation showed no characteristic absorption peaks for DOX. However, when the acidic solution with pH 4.0 was added into the DOX loaded nanoparticles, an absorption peak at 488 nm appeared, suggesting that DOX was successfully encapsulated into the nanocarriers and can be released by H^+^.

### 3.3. Multi-Responsive Drug Release from Complex Nanoparticles

**Figure 6:**
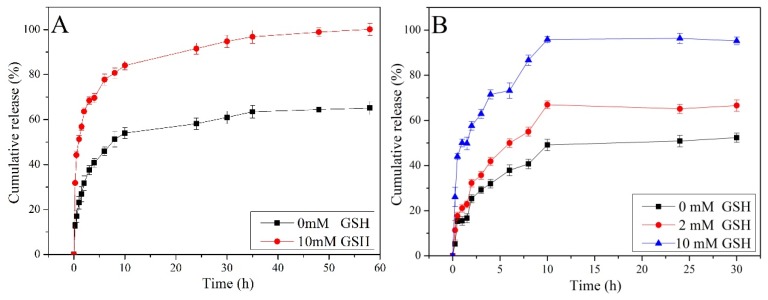
Release of DOX (**A**) and NAP (**B**) from nanocarriers at different concentrations of GSH (pH = 7.4, T = 25 °C).

Figure 6 illustrates the drug release behaviors of nanocarriers in the presence of different concentrations of GSH at 25 °C and pH 7.4. The results suggested the concentration of GSH affected the release behavior of the nanoparticles. First of all, the percentage of DOX and NAP released from the nanoparticles all reached 100% with 10 mM GSH. In contrast, the cumulative release of DOX and NAP without GSH was only about 60% and 50%. This can be attributed to the different fracture degree of the disulfide bond at various concentrations of reduced GSH [14]. Although there are no GSH to cut the disulfide bonds on the surfaces of the MSNs, some drugs are still cumulatively released from nanocarriers due to the uncompleted plugging of their mesopores or attached drugs on the surfaces of the MSNs.

**Figure 7:**
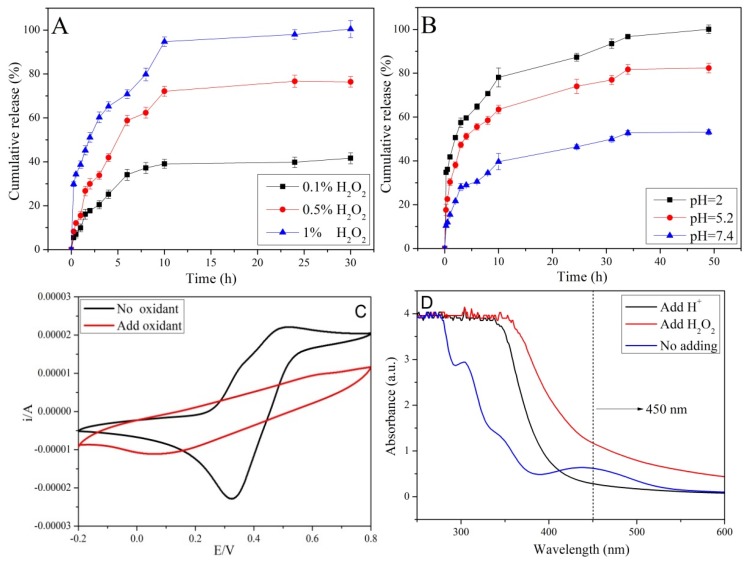
The release of NAP at different concentrations of H_2_O_2_ (**A**), release of DOX at different pH (**B**), the cyclic voltammetry curve (**C**), and the UV–Vis spectra of nanoparticles at different statuses (**D**).

In addition to the GSH stimulus-responsive ability, the nanocarriers can also be activated by H_2_O_2_ owing to the existing of an Fc group on the surface of the MSNs. Hydrophobic Fc was easily oxidized to hydrophilic Fc^+^ (Fc → Fc^+^ + e^−^) in the presence of H_2_O_2_, leading to a dissociation of the complexation between β-CD and Fc. β-CD-PNIPAM polymers are removed from the MSN-SS-Fc surface due to the electrostatic repulsion between hydrophobic β-CD and hydrophilic Fc^+^; as a result, drugs were released from the nanocarriers. The influence of H_2_O_2_ concentration on the drug release is presented in Figure 7A. When the concentration of H_2_O_2_ is increased from 0.1% to 1.0%, the cumulative release of NAP increased from 40% to approximately 100% over 30 h. In the first stage, the drugs were quickly released due to the degree of the disulfide bond fracture with different concentrations of GSH. Most of the drugs were released during the first 10 h, and the remaining drugs were slowly released because of a decrease of the concentration gradient. As reported in the literature, ferrocene is rapidly oxidized to ferrocene ions under acidic conditions, leading to the dissociation of nanocarriers and the rapid escape of drugs [30]. Given this, an additional experiment was carried out to further confirm the sensitivity of pH on the drug release (Figure 7B). The release of DOX was approximately 50% at pH 7.4. At pH 5.2 and 2.0, the accumulated drug release obviously increased to 80% and 100%, respectively. In a weak acidic environment, DOX was freely released from uncapped channels due to the opening gatekeeper of β-CD-PNIPAM and the decrease of electrostatic interactions between the MSNs and drugs [13,31].

The oxidation status of the iron was verified by CHI660E electrochemical analyzer (Chen Hua Instruments, Shanghai, China), as shown in Figure 7C. An anodic oxidation peak appeared in the range of 0.4–0.6 V when the potential was scanned in the positive direction, corresponding to the continuous oxidation of ferrocene near the electrode surface. When the potential was scanned in the negative direction, a cathode reduction peak appeared in the range of 0.3–0.4 V, which is associated with the continuous reduction of ferrocene cation. In the cyclic voltammetry curve after adding H_2_O_2_, there was only a cathodic reduction peak, indicating that most of the ferrous ions (Fe^2+^) in the ferrocene were oxidized to ferric ions (Fe^3+^). Ferric ions were reduced by an impressed current in the negative scanning process, resulting in a weak reduction peak. The status change of ferrocene under oxidants was also studied by UV–Vis. As shown in Figure 7D, the absorption peak disappeared at 450 nm after adding H_2_O_2_ or H^+^, indicating that ferrocene (Fc) was oxidized into the ferrocene ions (Fc^+^).

**Figure 8:**
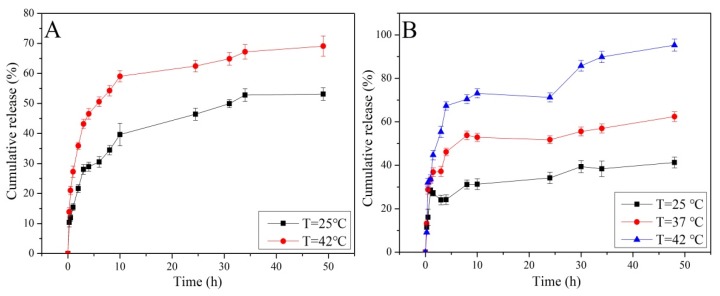
Release of DOX (**A**) and NAP (**B**) from nanocarriers at different temperatures.

Taking into consideration that the temperature around the tumor tissues is higher than that of normal tissues, the release behavior was investigated in PBS with different temperatures. As illustrated in Figure 8A, The cumulative release of DOX was about 50% at 25 °C and reached 70% at 42 °C within 50 h. For NAP, the cumulative release increased from 32% to 95% when the temperature increased from 25 to 42 °C. The increase of drug release with and an increase in temperatures ocurred because the extended PNIPAM chains collapsed and enlarged the gap of the nano-valve when the temperature was higher than the transformation point temperature. Comparing Figure 8B to Figure 8A, the release profile for NAP is sigmoid curves, and the cumulative release was higher than that of DOX. The most likely reason for this result was that the small molecular weight of NAP was easily captured by the cyclodextrin cavity and the long chains of PNIPAM via hydrogen bond interactions during their release from the MSN channels, which limited the diffusion process. This restriction was extremely noticeable at the first stage. As time goes on, these two effects gradually reach equilibrium, resulting in the sigmoid curves.

### 3.4. In Vitro Release Kinetics of Drugs

**Figure 9:**
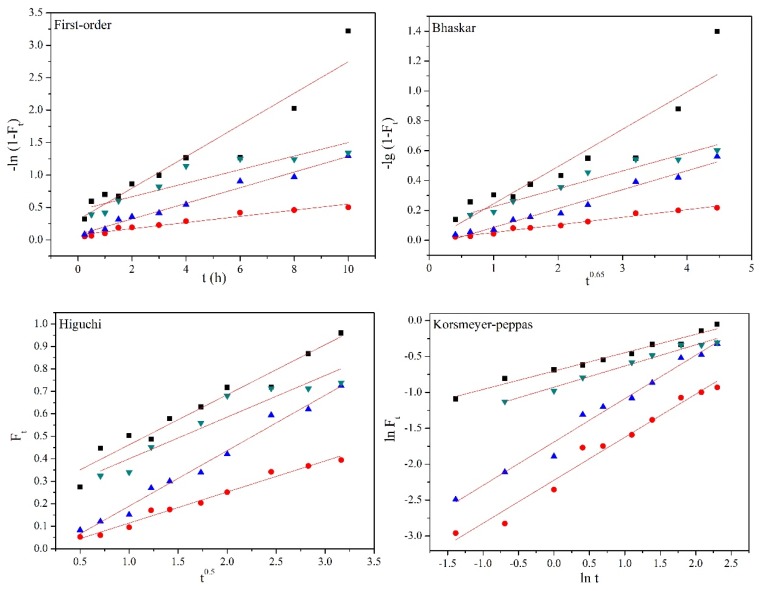
Kinetic plots of First-order, Bhaskar, Higuchi, and Korsmeyer–Peppas models for NAP release from MSN-SS-Fc@β-CD-PNIPAM nanoparticles.

The First-order, Bhaskar, Higuchi, and Korsmeyer-Peppas equations were used to explore the release mechanism of NAP from nanocarriers [32]. As shown in Table 3 and Figure 9, the high correlation coefficients (*R*^2^) indicates that the release mechanism was fitted with Higuchi and Korsmeyer–Peppas models under GSH. The release exponent *n* = 0.26 of the Korsmeyer–Peppas equation was used to describe the Fickian diffusion [33]. This diffusion mechanism was further supported by the highly correlated Higuchi model, and the release of NAP was not controlled by the weak interactions between NAP molecules and surface of the MSNs [34,35]. The high values of *R*^2^ for the Bhaskar, Higuchi, and Korsmeyer–Peppas models in the release medium of H_2_O_2_ demonstrated that the release mechanism was well fitted with these three kinetic models. The Bhaskar model corresponds to the controlled release behavior of intragranular diffusion, which may be caused by the weakening of the interactions between the NAP and MSNs. Similarly, it can be predicted that the elimination of complexation between ferrocene and cyclodextrin by H_2_O_2_ provides a shorter diffusion path length according than the Higuchi model. The release exponent *n* with 0.60 and 0.69 in the Korsmeyer–Peppas model indicates that the release behavior followed anomalous Fickian diffusion, mainly because some drugs were captured by the cavity of cyclodextrin during the release process. Thus, the diffusion of NAP and the removal of cyclodextrin simultaneously occurred with the oxidation of H_2_O_2_. The *R*^2^ of the Korsmeyer–Peppas model was found to be higher than 0.960 and *n* = 0.29 in the release medium of 42 °C, revealing the Fickian diffusion-controlled mechanism. The rapid NAP release was because the collapse of temperature-sensitive PNIPAM increased the gap between macromolecular chains and shortened the release path from the nanocarriers.

## 4. Conclusion

In summary, a temperature, GSH, and H_2_O_2_ multi-responsive drug delivery system based on a β-CD-PNIPAM coating MSN-SS-Fc was successfully designed through host–guest interactions. The release profiles of the nanocarriers indicates that the drugs were quickly released in low pH with H_2_O_2_ environments due to the protonation of Fc. The disulfide bonds of the nanocarriers were fractured by GSH, with which approximately 100% of the drugs were released in the presence of 10 mM GSH. The drug release from nanocarriers was also triggered by an increase of temperature due to the external temperature-sensitive PNIPAM chain coating on the surface of the MSN-SS-Fc. These multi-stimuli sensitive nanocarriers appear to be promising for the development of a platform in drug delivery applications.

## Figures and Tables

**Table 1:** Elemental analysis data for MSNs, MSN-SH, and MSN-SS-Fc.

Samples	C%	H%	S%	N%
MSNs	1.69	0.71	0.79	0.19
MSN-SH	6.23	1.40	3.58	0.16
MSN-SS-Fc	7.77	1.86	3.72	1.37

**Table 2:** The drug-loading content (DLC%) and entrapment efficiency (EE%) of the nanoparticles for DOX and NAP.

*m*_0_/*m*^a^ (%)	DOX		NAP	
	DLC (%)	EE (%)	DLC (%)	EE (%)
20	12.01	68.27	11.76	49.58
40	23.22	75.63	20.00	62.50
60	31.56	76.87	27.69	71.23
80	38.28	77.85	32.12	70.76
100	41.81	71.86	34.64	66.26

^a^*m*_0_ is the weight of drug dosage; *m* is the weight of nanoparticles.

**Table 3:** Kinetic parameters of drug release in different mediums.

Release Medium	Firs-order	Bhaskar	Higuchi	Korsmeyer–Peppas	
	*R* ^2^	*R* ^2^	*R* ^2^	*R* ^2^	*n*
10 mM GSH	0.899	0.841	0.950	0.972	0.26
0.1% H_2_O_2_	0.946	0.981	0.980	0.971	0.60
0.5% H_2_O_2_	0.983	0.973	0.984	0.982	0.69
42 °C	0.823	0.945	0.886	0.980	0.29
